# Evolving from Laboratory Toys towards Life-Savers: Small-Scale Magnetic Robotic Systems with Medical Imaging Modalities

**DOI:** 10.3390/mi12111310

**Published:** 2021-10-26

**Authors:** Jiachen Zhang

**Affiliations:** Department of Biomedical Engineering, City University of Hong Kong, Hong Kong, China; jzhang.bme@cityu.edu.hk

**Keywords:** small-scale robotics, magnetic control, medical imaging, minimally invasive healthcare

## Abstract

Small-scale magnetic robots are remotely actuated and controlled by an externally applied magnetic field. These robots have a characteristic size ranging from several millimetres down to a few nanometres. They are often untethered in order to access constrained and hard-to-reach space buried deep in human body. Thus, they promise to bring revolutionary improvement to minimally invasive diagnostics and therapeutics. However, existing research is still mostly limited to scenarios in over-simplified laboratory environment with unrealistic working conditions. Further advancement of this field demands researchers to consider complex unstructured biological workspace. In order to deliver its promised potentials, next-generation small-scale magnetic robotic systems need to address the constraints and meet the demands of real-world clinical tasks. In particular, integrating medical imaging modalities into the robotic systems is a critical step in their evolution from laboratory toys towards potential life-savers. This review discusses the recent efforts made in this direction to push small-scale magnetic robots towards genuine biomedical applications. This review examines the accomplishment achieved so far and sheds light on the open challenges. It is hoped that this review can offer a perspective on how next-generation robotic systems can not only effectively integrate medical imaging methods, but also take full advantage of the imaging equipments to enable additional functionalities.

## 1. Introduction

In the past several decades, small-scale robotics has been attracting an increasing amount of attention from researchers, entrepreneurs, and healthcare professionals. These miniature devices have the potential to access hard-to-reach regions in human bodies and perform unprecedented medical procedures in a minimally invasive manner [1,2,3,4,5,6,7,8]. In early 2020, the World Health Organization (WHO) declared the novel coronavirus 2019 (COVID-19) as a pandemic. Since then, the characteristic of these robots being remotely controlled is further highlighted and becomes particularly attractive. It reduces the cut in operations to minimize contamination and allows healthcare professionals to safely distance from patients during medical procedures for infection control. In addition, with the rapid deployment of 5G network, these robots also offers the possibility of long distance diagnosis and treatment via teleoperation, when travelling is costly, inconvenient, and even dangerous during a global pandemic. In the peri- and post-pandemic eras, these miniature robots promise to meet the ever more volatile healthcare challenges. They aim to bring a more personalized, more versatile, and less invasive healthcare solution.

Small-scale magnetic robotics emerged as an interdisciplinary research field several decades ago and quickly gained momentum in advancement [9,10,11,12,13,14,15,16,17,18,19,20]. Magnetic field excels other control approaches due to its abilities of simultaneously generating both forces and torques on remote objects while safely penetrating biological substances. Pioneering endeavours are being made to push this field towards commercialization and adoption by the healthcare systems. However, existing studies are still mostly limited to laboratory environments with unrealistically simplified working conditions. This excess simplification damages the fidelity in mimicking the biological workspace inside human body. And the resultant robotic systems can only serve as proof-of-concept units without real-world applicability. For next-generation small-scale magnetic robotic systems, it is compulsory to enhance their real-world relevance and evolve beyond laboratory environments. Recognizing this trend, recent studies have started to consider clinical constraints and requirements. Focus has also been put on the feasibility of the developed robotic systems in the targeted real-world workspace. Particularly, imaging and sensing using medical imaging modalities are been intensively investigated. Because they are indispensable for these robotic systems to work safely and effectively inside human body. They are also the cornerstone for teleoperation and semi-/full-automation control of these robots. Most early studies employed optical camera or even naked eye as the primary imaging modalities. But direct line-of-sight does not exist in the targeted clinical workspace of these miniature robots. When these robots go inside human body and work within deep-buried cavities and ducts, medical imaging modalities have to be employed, such as magnetic resonance imaging (MRI), ultrasound (US), X-ray, fluorescence imaging, and others.

It is beneficial to clarify the robot definition in the context of small-scale robotics. As individual device gets smaller, it becomes simpler in materials and structures. And more capabilities that were used to be accomplished by onboard components are now fulfilled by off-board peripherals and supporting equipments. From a few millimetres down to a few nanometres, these devices serve more like one [21,22,23,24,25,26] or a swarm of [27,28,29,30,31] miniature end-effectors of a larger robotic system. For example, Zhang et al. reported a 3.5 mm tip-to-tip long microgripper that automatically recognised and transported various microobjects in 3D [23]. They are referred to as “robots” with an implication that they function as a part of a robotic system towards robotic applications. With the definition clarified, this review discusses some exemplar recent efforts in employing medical imaging modalities to render the developed small-scale magnetic robotic systems more applicable to real-world biomedical tasks. This review does not include an exhaustive list of studies, but sets its priority to be informative while remaining concise. It is hoped that this review will inspire fellow researchers by pointing out future directions and open challenges of integrating medical imaging modalities in the following studies that aim at realistic biomedical applications.

## 2. Conventional Imaging Setup for Small-Scale Magnetic Robots

**Figure 1:**
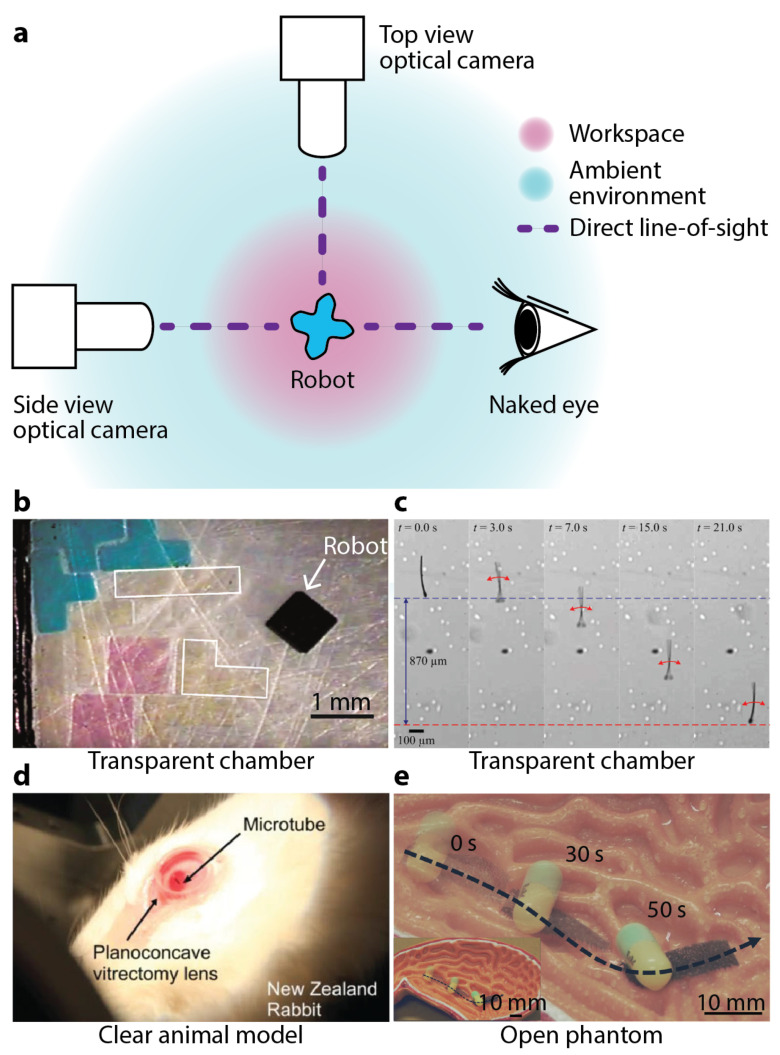
Conventional imaging setup for small-scale magnetic robots in laboratory environments. (**a**) Schematic illustration of a classic configuration in laboratory tests of small-scale magnetic robots. (**b**) Robot in a transparent chamber: A robot coded various material composition on a 2D substrate. Reproduced with permission from [26]. Copyright © 2014 Nature Publishing Group. (**c**) Robot in a transparent chamber: A sperm-like robot swam inside a transparent chamber. Reproduced with permission from [32]. Copyright © 2014 AIP Publishing. (**d**) Robot in a clear organ: A microrobot moving inside the vitreous humor of an anesthetized New Zealand rabbit. Reproduced with permission from [33]. Copyright © 2014 WILEY-VCH Verlag GmbH & Co. KGaA, Weinheim. (**e**) Robot in an open phantom: A millimetre-scale soft magnetic robot carried a drug pill in an open stomach model under wet environment. Reproduced with permission under a Creative Commons Attribution 4.0 International License [34].

With a short history of only several decades, studies in small-scale magnetic robotics have hitherto limited themselves to simplified laboratory environments and proof-of-concept demonstrations. In these pioneering investigations, direct line-of-sight was maintained to easily track the robots within different workspace. Optical cameras and sometimes even naked eyes were employed as the primary or only imaging modalities, see Figure 1a for a schematic of one common setup. The optical images of the robots are easy to obtain and also are intuitive for human operators and computer controllers to process and understand. Moreover, an abundance of image processing software and packages are readily accessible and well-suited for optical images, conveniently extracting information to feed human operators or (semi)autonomous controllers.

Not willing to forsake the aforementioned convenience, researchers have been trying to maintain such a direct line-of-sight in as many cases as possible. Many experiments, especially the preliminary ones, of small-scale magnetic robots are performed in air [35,36,37,38], at an interface [39,40,41,42,43,44,45], or in an aqueous medium inside a transparent container, such as a Petri dish, a beaker, or a tube [32,46,47,48,49,50,51,52,53,54,55]. For example, Tasoglu et al. used an untethered magnetic microrobot to code three-dimensional (3D) materials with tunable structural, morphological, and chemical features [26]. A top-view camera observed the robot working on a two-dimensional (2D) substrate, see Figure 1b. Khalil et al. employed a top-view optical camera to observe the sperm-like microrobot they proposed [32], see Figure 1c. This 322 μm long robot was named as the MagnetoSperm. And it exhibited an average speed speed of 158 ± 32 μm/s when being actuated by an alternating magnetic field of 45 Hz with an amplitude less than 5 mT. Diller et al. used a top-view optical camera to observe a swimming millimetre-scale magnetic sheet at water surface [44]. The sheet bore a sinusoidal magnetization profile and exhibited continuous undulatory deformation along its body in a rotating magnetic field. With the convenience provided by the direct line-of-sight, autonomous controllers have been developed for these small-scale magnetic robots. For example, Pawashe et al. developed an autonomous manipulation strategy for an untethered magnetic microrobot on a 2D surface [56]. Positions of the microrobots and also the target objects were tracked in real-time using an optical camera. Ryan et al. controlled a microrobot to follow pre-defined 3D paths in a liquid environment using a configuration of eight permanent magnets based on real-time optical feedback [57]. Huang et al. proposed a closed-loop control method for swimming magnetic microrobot for 3D arbitrary path following using visual servoing [58]. Dong et al. developed a position estimator-based motion controller for magnetic microrobots working inside a simulated vascular structure [59].

Even when medical phantoms or animal models were used for realistic biomedical working environments, open [34,60,61,62] and clear phantoms [63,64,65,66,67] and clear animal model such as eyeball [33,68] were chosen to avoid obstructing the direct line-of-sight. In 2015, Chatzipirpiridis et al. deployed a needle-like microrobot into the vitreous humor of a living rabbit eye [33], see Figure 1d. An optical camera observed the locomotion of this robot inside the vitreous humor. In 2017, Son et al. designed a magnetically actuated soft capsule endoscope robot for fine-needle aspiration biopsy (B-MASCE) in the upper gastrointestinal (GI) tract [62]. Experiments were performed inside an open anatomical human stomach model. In 2018, Lu et al. reported an untethered soft millimetre-scale robot with multiple tapered soft feet architecture [34]. Experiment showed its drug transport capability within an open stomach phantom, see Figure 1e. In 2019, Kim et al. reported a magnetically guided soft continuum robot to navigate within a 3D cerebrovascular phantom network, which was filled with a blood analog [65]. The whole setup consisting of the network and the blood analog was transparent and visually observed by the operator in operation. In 2020, Zhang et al. used two optical cameras to observe a magnetically-driven screw robot moving inside a clear agar gel tissue phantom [64]. One of the camera was mounted above the workspace. And the other one was mounted in front of it. In 2021, Dong et al. reported a magnetic microswarm made up of porous Fe${}_{3}$O${}_{4}$ mesoparticles for biofilm disruption [63]. Biofilms are 3D collections of microorganisms on the surfaces of organs such as teeth, bone, and urethra. The authors placed biofilm inside a clear U-shaped tube, and observed it before and after the microswarm treatment. Manamanchaiyaporn et al. developed a magnetic nanoparticle swarm to induce hydrodynamic effect to capture tissue plasminogen activator (t-PA) for thrombolytic therapy [69]. An optical camera was used to observe the swarm within a transparent tubular environment. In these studies, direct line-of-sight was maintained and the robots were visually observed by the operators in real-time, without implementing any medical imaging modalities.

Although the optical imaging approach is widely adopted, only using this technique compromises the clinical relevance of the corresponding small-scale robotic systems. Because direct line-of-sight is unavailable for most clinical scenarios, especially for minimally invasive diagnostic and therapeutic tasks that motivate small-scale robotics in the first place. As a result, researchers have started to restrain the usage of optical imaging to only early stage proof-of-concept demonstrations. Instead, they actively seek to deploy more realistic and clinically relevant imaging modalities in next-generation small-scale magnetic robotic systems.

## 3. Medical Imaging Modalities

**Figure 2:**
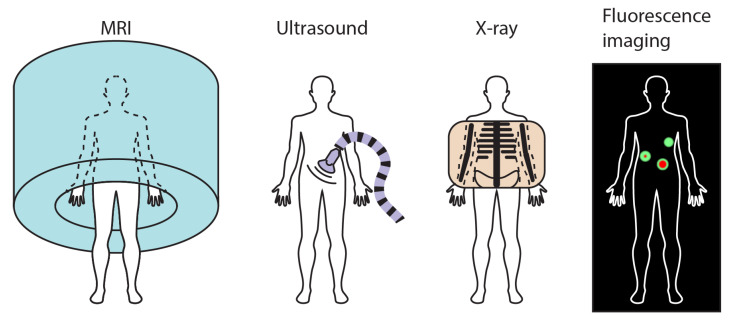
Schematics of four exemplar medical imaging modalities that are relevant with small-scale magnetic robots. They are magnetic resonance imaging (MRI), ultrasound (US), X-ray, and fluorescence imaging.

Imaging biology tissues and tracking markers are pivotal capabilities in clinical environments. They provide healthcare practitioners with static and dynamic information beyond direct visual observations to assist in disease prevention, diagnosis, and therapy [70]. A wide plethora of medical imaging modalities have been developed and deployed to facilitate the identification of different medical complications. Some widely adopted ones include MRI, US, X-ray, and fluorescence imaging, which are schematically illustrated in Figure 2. Each modality possesses attributes that make it uniquely useful for some specific kinds of disease and abnormality. Considering the countless kinds of diseases and abnormalities affecting different parts of the human body, there is no single imaging method that dominates by providing better performance in all cases.

Though these imaging modalities are common in clinical workspace, they require dedicated and specialized equipments which are often expensive to purchase, setup, operate, and maintain within research laboratories hosted by universities and institutes. The resultant medical images are different with the optical ones that people are used to. And professional training is required for proper interpretations and information extraction from medical images. In addition, as all these imaging modalities were developed prior to the emergence of small-scale magnetic robotics, they are not inherently compatible with these novel robotic systems. Comprehensive evaluations and validations of compatibility and safety are needed before integrating them together. The steep upfront monetary investment leads to the scarcity of these imaging modalities in research communities. For the privileged researchers who have access to these modalities, the operational cost, expertise requirement, and prerequisite compatibility and safety studies discourage them from adopting these modalities in their investigations of small-scale magnetic robots. Although clinical imaging modalities have the aforementioned inhibitive features, researchers still have started to make efforts towards the integration of them in the next-generation of small-scale magnetic robotic systems. Because these imaging modalities constitute an indispensable portion of future robotic systems applied in real-world biomedical applications, where direct line-of-sight is unavailable.

## 4. Integrating Medical Imaging Modalities in Small-Scale Magnetic Robots

This section reviews some exemplar studies that take advantages of medical imaging modalities to enhance the relevance of the developed small-scale magnetic robotic systems with real-world biomedical applications. The scope of this section is intentionally limited to only the studies involving MRI, US, X-ray, or fluorescence imaging.

### 4.1. Small-Scale Magnetic Robotic Systems with MRI

MRI is a versatile imaging technique to generate images of human bodies, especially soft tissues. It uses a strong uniform axial magnetic field, magnetic field gradients, and radio waves. The strong axial magnetic field has a magnitude of several teslas. And it poses challenging constraints on the use of ferromagnetic materials as well as conductive cables. During MRI imaging, patients are confined to a long and narrow central chamber with loud noise that could range from 65 to 130 decibels, limiting the appropriate time period of operation. Thus, small-scale magnetic robots have to work in a strong constant magnetic field, with all necessary peripherals fitting inside the limited space and operation finished by a relatively short period of time. But at the same time, MRI machines have the potential to carry out imaging and actuation of small-scale magnetic robots without requiring additional setups, assuming the magnetic robots could take advantages of the magnetic field generated by the MRI machines.

**Figure 3:**
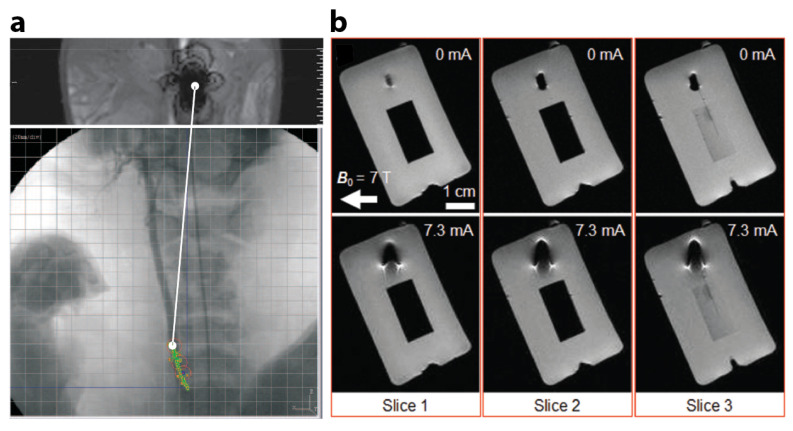
Exemplar studies of small-scale magnetic robots with integrated MRI modality. (**a**) Distorted MRI image created by a 1.5 mm-diameter chrome steel sphere, which was tracked over a pre-acquired X-ray image while being controlled at 24 Hz in the carotid artery of a living swine along the waypoints. Reproduced from [73] under STM Permissions Guidelines. (**b**) Three different MR image slices of the nonoperated (off, 0 mA) actuator showing no image artifact and operated actuator showing beam bending and generated image artifacts inside a preclinical MRI scanner. Reproduced with permission under a Creative Commons CC BY license [74].

Efforts have been made to use MRI machines to obtain real-time position information of small-scale magnetic robots. In 2008, Felfoul et al. developed a 3D magnetic resonance (MR)-tracking method specifically for small-scale robots with a ferromagnetic core [71]. In vitro and in vivo experiments validated its feasibility in tortuous phantom and in animal models. Experimentally obtained MR images showed in vivo movement of a catheter tip carrying a ferromagnetic sphere in a 35 kg domestic pig. In 2009, Martel et al. reported a medical interventional platform using MRI for feedback information [72,73]. The platform used a magnetotactic bacterium as an integrated small-scale robotic system. Figure 3a shows an experimental MR image of the distortion due to the 1.5 mm-diameter chrome steel sphere used in the validation phase. The sphere was controlled at 24 Hz in the carotid artery of a living swine. An optical pre-acquired X-ray image was provided below the MR image for additional physiological information.

In 2017, Yan et al. reported biohybrid small-scale robots with helical shapes for MRI-guided therapy [75]. In vivo MRI tracked a swarm of such robots inside rodent stomachs. Experimental MR images compared the distributions of a swarm of robots subject to magnetic actuation and steering for 5 and 10 min before MRI. Susceptibility artifacts close to the subcutaneous tissue were observed which implied the existence of high-concentration regions (rich in the proposed biohybrid robots) nearby. In 2021, Zhang et al. showed in vivo MR imaging of mice after injection of a swarm of neutrophil-based microrobot, which the authors referred to as neutrobot [76]. These neutrobots can cross the blood-brain barrier and actively deliver cargoes to malignant glioma in vivo, guided by a rotating magnetic field.

Since MRI machines generate both uniform magnetic field as well as magnetic field gradient, it is preferable to develop small-scale magnetic robots that can not only be imaged but also be actuated and controlled by MRI machines, obviating the need for additional magnetic field generation setups. Though at any instance, only one of the functions, i.e., either sensing or actuation, can be executed by the same MRI machine. In 2015, Lalande et al. took advantages of the presence of a strong magnetic field in a MRI machine and successfully demonstrated in vivo magnetic steering of a ferromagnetic guidewire in a MRI system equipped with additional gradient coils for selective catheterization of renal arteries on rabbits [77]. Also towards this end, Erin et al. proposed a robot design that can rotate in 3D in MRI-like magnetic environments using commercially available MRI gradient coil in 2019 [78]. In 2020, Mutlu et al from the same research group presented a Lorentz force-based electromagnetic actuator with MRI compatibility which is optically powered via a solar cell [74]. The actuator has a dimension of 2.5-by-2.5-by-3.0 mm${}^{2}$. Energy is delivered to the actuator using optical fibres that shine laser light on the solar cell. Its modular design enables the combination of a number of actuators for extended functionalities. MR observations of the actuator are presented in Figure 3b. Continuing this effort, in 2021, Erin et al proposed a capsule robot that is not only imaged but also powered by a MRI machine [79,80]. The robot demonstrated steerable navigation, medical function, and MRI tracking capabilities inside a preclinical small-animal MRI machine, which wirelessly powered and monitored the robot. A MRI machine provided both imaging and actuation for a small-scale magnetic robot in real-time.

Besides experimental explorations, theoretical and computational research has also been performed to construct a plan and control strategies as well as simulation environment for microrobots using a MRI machine [81,82]. In 2010, Belharet et al. constructed a system software architecture for ferromagnetic microrobot working with a MRI system [82]. The authors validated the proposed imaging processing and control algorithms using simulation results. In 2020, Tiryaki et al. presented a dynamic simulation environment for MRI-based tracking and actuation of untethered magnetic microrobots [81].

The configurations and working conditions of MRI machines pose severe constraints on the deployment of small-scale magnetic robots. But at the same time, the increasing accessibility and popularity of MRI machines in healthcare keeps attracting researchers. In addition, MRI is a widely applied and thoroughly investigated medical imaging modality. It poses no safety concerns for patients assuming standard precautions and operational procedures are followed. As a result, it is a particularly intriguing promise of fusing imaging and controlling systems into one MRI setup for better reliability and less complexity. Imaging requires a strong axial magnetic field and short pulses of radio frequency (RF) fields. While actuation requires quasi-static magnetic fields at various directions with smaller magnitude compared with the axial magnetic field for imaging. As the magnetic fields used for imaging and actuation are different, it exists a possibility of developing a multiplex of magnetic field sequence to conduct both tasks. One challenge is that the strong axial magnetic field required for imaging is always present in the workspace and override the effects of any quasi-static magnetic fields used for actuation. Next-generation robotic systems are being designed to utilize an MRI machine to its full potential. Table 1 summarizes the reviewed studies in this section.

### 4.2. Small-Scale Magnetic Robotic Systems with US Imaging

US imaging is widely used in clinical scenarios to visualize muscles, tendons, and other internal organs of human body [83]. It stands out from other candidate medical imaging modalities due to its relatively simple setup, high versatility, good portability, and low operational and maintenance cost. It is an extremely popular medical imaging modality in clinical scenarios with better accessibility than other. The handhold US probe can be easily placed at desired configurations and adjusted in operation.

**Figure 4:**
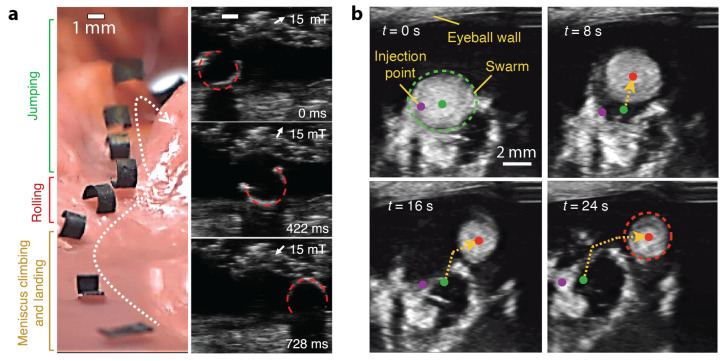
Exemplar small-scale magnetic robots imaged by US. (**a**) A millimetre-scale soft magnetic robot moved inside a phantom made by ex vivo chicken tissue. Reproduced with permission from [84]. Copyright © 2018, Springer Nature. (**b**) Navigated locomotion of a swarm of small-scale magnetic robots inside a bovine eyeball with US imaging feedback. Reproduced with permission under a Creative Commons Attribution 4.0 International License [86].

In 2018, Hu et al. demonstrated that a millimetre-scale magnetic sheet robot was wirelessly modulated to move inside a concealed areas of an ex vivo chicken tissue [84]. Figure 4a shows this shape-morphing robot moving inside a phantom made of chicken meat under visual and US observation side-by-side. In 2019, Ren et al. from the same group reported a millimetre-scale jellyfish robot that patched the target surface under the guidance of US imaging inside a bladder phantom [85]. Yu et al. experimentally demonstrated the navigated locomotion process of a swarm of nanoparticles inside a bovine eyeball with US imaging feedback, see Figure 4b [86].

In 2020, Wang et al. reported a reversible anchoring robot that worked within a tubular structures under the guidance of real-time US images [87]. The soft polyurethane elastomer-based robot is 7.5 mm in diameter and 17.8 mm in length. It anchored itself stably at a target position without consistent input, and released on-demand to locomote to another position. The US probe was mounted at the tip of a robotic arm to perform pre-programmed scanning procedure with good repeatability. Niedert et al. showed a tumbling small-scale robot that moves in ex vivo, in vitro, in situ, and in vivo conditions under real-time US guidance [88]. In 2021, Wang et al. developed a complete system for magnetic navigation of biohybrid small-scale robots with rapid endoluminal delivery and imaging [89]. This system is named as endoscopy-assisted magnetic actuation with dual imaging system (EMADIS) and offers a full clinical imaging technique-based therapeutic/intervention system. From the same group, Wang et al. reported a strategy to navigate a nanoparticle microswarm in real-time under ultrasound Doppler imaging guidance for active endovascular delivery [90].

US has become one of the most accessible and popular medical imaging modality for researchers in small-scale magnetic robotics. It has also been used to provide acoustic energy as the primary or additional input for small-scale robots. For example, Youssefi et al. reported a dual micromanipulation system for small-scale robots to achieve 3D, contactless, and semi-autonomous micromanipulation in 2019 [91]. Robots ranging from 3 mm down to 300 μm were acoustically levitated within the workspace and controlled by an ambient magnetic field. In 2020, Darmawan et al. demonstrated magnetically controlled helical small-scale robots that release onboard drugs under ultrasound stimulation [92]. Thus, there exists an exciting opportunity to utilize US for not only imaging but also actuation in next-generation small-scale robotic systems. Similar with MRI, US does not pose safety concerns for the patients. But at the same time, it is much more versatile and accessible than MRI. Thus, US is the most desirable medical imaging modality to be utilized in small-scale robotic systems. Table 2 summarizes the reviewed studies in this section.

### 4.3. Small-Scale Magnetic Robotic Systems with X-ray Imaging

X-ray imaging modality employs ionizing radiation to generate images of human bodies. When (a part of) human body is sandwiched between a radiation source and sensor, different biological substances of the body block the radiation to various extents, resulting in different signal strength received by the sensor. X-ray is widely used in clinical workspace and includes different specific techniques and procedures tailored for different purposes. Examples include radiography, computed tomography (CT), and X-ray fluoroscopy. To roughly differentiate these three techniques from each other in terms of their usage, radiography captures single still images, CT provides a reconstruction using many cross-sectional images, while X-ray fluoroscopy produces a continuous X-ray image. But recent advancement of these technologies has blurred their distinctions, and the overlap of their respective functionalities grows larger. Although X-ray imaging modalities are widely deployed and frequently utilized in clinical environments, the exposure to radiation leads to potential risks, such as injuries to the skin and underlying tissues (“burns”) shortly after the procedure and radiation-induced cancers occurring some time later. To minimize these risks, X-ray imaging is always performed with the lowest acceptable exposure for the shortest time necessary.

**Figure 5:**
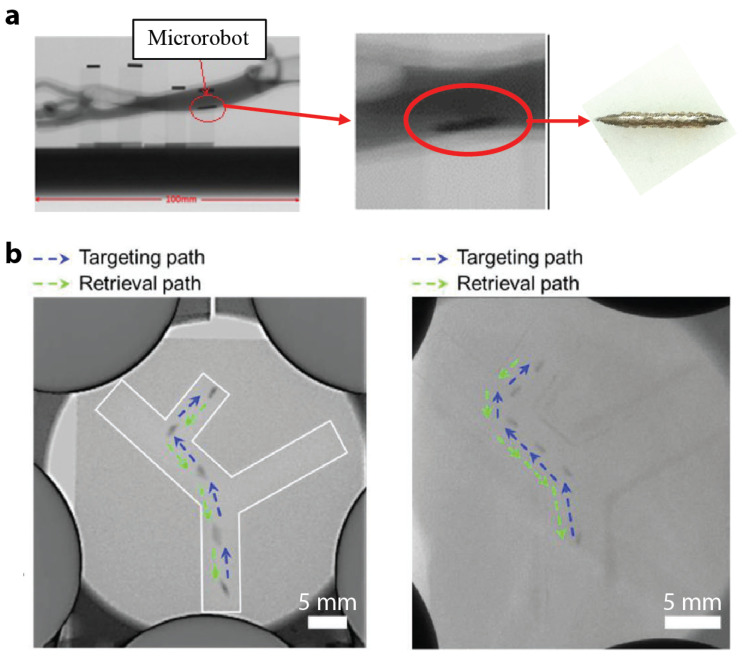
Exemplar studies of small-scale magnetic robotic systems with fluorescence imaging modalities. (**a**) X-ray images of an intravascular microrobot. Reproduced with permission from [93]. Copyright © 2018, CARS. (**b**) Time-lapse X-ray images of a microrobot moving in the 2D vessel phantom with and without a thick layer of pork meat on top of the channel. The white contour is the channel outline. Reproduced with permission from [94]. Copyright © 2021 Wiley-VCH GmbH.

In 2018, Nguyen et al. proposed a real-time position and spatial orientation tracking method for a millimetre-scale bullet-shaped robot using a principle component analysis and an X-ray reconstruction [93]. The developed method utilized a biplane X-ray imaging system consisting of two perpendicular X-ray devices. Figure 5a shows an example of the captured image using this system alongside with an optical image of the robot. In 2020, Yang et al. presented radiology images of a magnetic-skin (M-skin) capsule steered in a stomach of a rabbit with a controlled O trajectory [61]. Imaging small-scale robots with X-ray is challenging because of the low density of these robots and the high permeability of X-rays for small objects. In 2021, Nguyen et al. proposed a magnetically guided rod-shaped small-scale robot for targeted drug delivery under real-time X-ray imaging [94]. The robot was loaded with an X-ray contrast agent, i.e., Lipiodol, to improve the imaging outcome. Experimental results demonstrated that the robot reached the targeted site via autonomous navigation with real-time X-ray imaging. Then, the robot released its loaded drugs through near-infrared radiation (NIR) triggering, and was recovered through a microrobot injector. Figure 5b shows time-lapse X-ray images capturing the robot motion in a 2D vessel phantom without and with a thick layer of pork meat on top of the channel.

X-ray is a convenient choice to image small-scale magnetic robots for in vitro, ex vivo, and in vivo biological experiments performed. However, as it involves ionizing radiation by its nature, the safety of its wide deployment in next-generation robotic systems warrants further investigations. Established medical guideline such as “As Low as Reasonably Achievable” (ALARA) requires careful evaluations been performed to compare the benefits with the risks associated with X-ray imaging. Table 3 summarizes the reviewed studies in this section.

### 4.4. Small-Scale Magnetic Robotic Systems with Fluorescence Imaging

Although they may sound similar, fluorescent imaging is different with X-ray fluoroscopy. Fluorescence imaging utilizes fluorophores and a fluorescence-detecting imaging system, avoiding the exposure to radiation and thus ameliorating the safety concern [95]. Fluorophores are microscopic molecules that emit light of longer wavelengths (thus lower energy) when being activated by light at specific wavelengths (the Stokes shift). Fluorophores can be natural substances as well as synthetic polymers. Fluorescence imaging circumvents the limitation of X-ray fluoroscopy and offers safe, cost-efficient, and real-time intraoperative visualization of anatomical structures highlighted by fluorophores.

**Figure 6:**
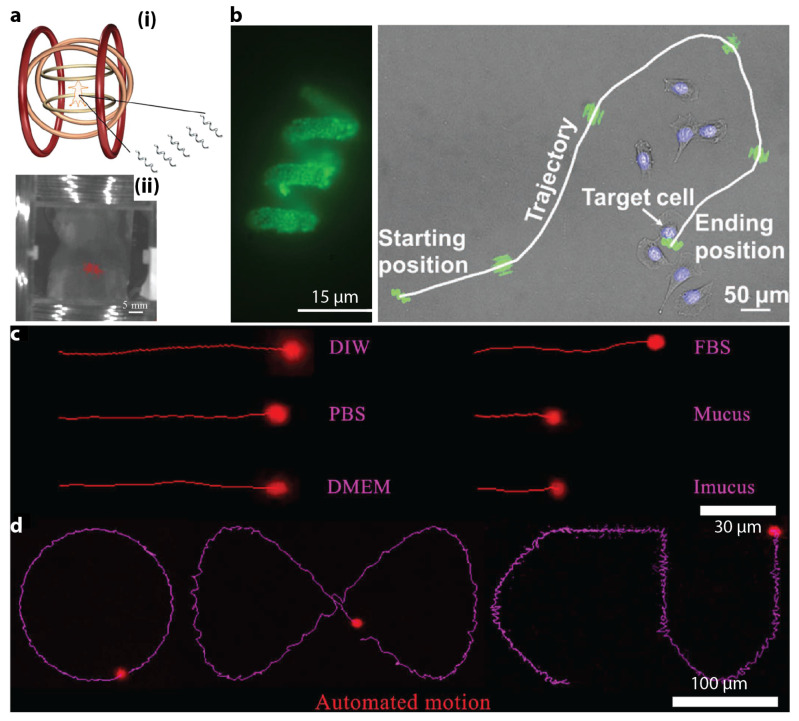
Exemplar studies of small-scale magnetic robotic systems with fluorescence imaging. (**a**) Fluorescence imaging of the swimming of a swarm of f-ABFs in vivo. Reproduced with permission from [96]. Copyright © 2015 WILEY-VCH Verlag GmbH & Co. KGaA, Weinheim. (**b**) A small-scale helical swimmer was observed using fluorescence imaging. Microscopy image shows the movement of a swimmer along a trajectory to target at a single cell. Reproduced with permission from [98]. Copyright © 2019 WILEY-VCH Verlag GmbH & Co. KGaA, Weinheim. (**c**) Fluorescence motion trajectories of the microrobots in DIW, PBS, DMEM, FBS, mucus, and intestinal mucus (Imucus) in a rotating magnetic field within 6 seconds (10 mT at 4 Hz). Reproduced with permission from AAAS [99]. (**d**) Fluorescence trajectories of the autonomous navigation of the microrobots in DIW according to predefined paths. Reproduced with permission from AAAS [99].

In 2015, Servant et al. employed real-time fluorescence imaging to observe a swarm of micron-scale functionalized artificial bacterial flagella (f-ABFs) [96]. The f-ABFs were controlled to swim in the intra peritoneal cavity of a Balb-C mouse. Figure 6a(i) illustrates the setup for the in vivo experiment. A fluorescent image of an anesthetized 4-week old Balb-C mouse inside the setup is shown in Figure 6a(ii), in which the red spots represent the fluorescence signal of the injected f-ABFs. A large enough amount of f-ABFs were deployed to make the fluorescence signal strong enough for detection. The study reported by Yan et al. in 2017 not only used MRI imaging, but also took advantages of fluorescence imaging for in vivo test of biohybrid small-scale robots with helical shapes [75]. In 2018, Li et al. proposed a micron-scale burr-like porous spherical robot that carried and delivered targeted cells in vivo [97]. The robot was coated with Ni to be driven by magnetic field gradient and also coated with Ti for biocompatibility. A swarm of such robots carrying HeLa green fluorescence protein-positive (GFP${}^{+}$) cells, which were tumorigenic cells that can generate tumour in weeks, were injected subcutaneously into the left dorsum of a nude mouse. Fluorescence imaging results during 4 weeks of cultivation indicated that a tumour was formed due to the injected HeLa cells.

In 2019, Wang et al. reported magnetic helical microstructures that were controlled to swim along pre-defined trajectories under the control of rotational magnetic fields for single-cell targeting [98]. Experimental fluorescence images are shown in Figure 6b. Zhang et al. described the use of fluorescence magnetic spore-based microrobots (FMSMs) as a highly efficient mobile sensing platform for the detection of toxins secreted by Clostridium difficile (C. diff) that were present in patients’ stool [99]. Such microrobots were tested in biological environments under real-time tracking of fluorescence imaging. In Figure 6c, the microrobots moved within external media, even real biological samples (mucus). In Figure 6d, the microrobots showed a more precisely controllable fluorescence motion with automatic and continuous operation along predefined trajectories. Jeon et al. reported the usage of magnetic microrobot as a platform for stem cell transplantation [100]. In vivo experiments were conducted to transport mcirorobots carrying hTMSCs in an athymic BALB/c nude mouse (6 to 8 weeks of age) under the actuation of an externally applied magnetic field. Successful transportation was verified using in vivo fluorescence imaging. In 2020, Yasa et al. captured the fluorescence images showing live actin protrusion during the crawling of a double-helical microswimmer [101].

Fluorescence imaging is popular in small-scale magnetic robotic systems. Different with other imaging modalities, it lacks the capability to reveal hidden features of the biological environment surrounding the robots. Thus, it cannot help the robots navigate within complex and tortuous environments buried deep in the body. Instead, it is often used to reveal the position of one [98] or a swarm of robots [97] with respect to the whole body to validate targeted delivery. As fluorophores can be toxic, sufficient investigations must be carried out before their administration to patients [102]. Table 4 summarizes the reviewed studies in this section.

## 5. Outlook

The past several decades witnessed the emergence and rapid advancement of small-scale magnetic robotic systems. Pioneering explorations carried out by researchers across the globe have presented auspicious preliminary results. Researchers, engineers, and entrepreneurs are joining efforts to push this field forwards and determined to deliver its full potential. These robots have started to evolve from laboratory toys towards the promised life-savers in biomedical tasks, especially in the peri- and post-pandemic eras. To realize a such transition, next-generation small-scale magnetic robots demand more than proof-of-concept demonstrations in simplified ideal environments. Instead, application-oriented robots need to be developed for unstructured biological workspace under real-time guidance of medical imaging modalities. The success of this transition will warrant a long-term prosperity of this field and lead to far-reaching societal and economic impact.

Imaging small-scale robots using medical imaging modalities is challenging. Concerted and also independent efforts are being made in the research community to explore various approaches and strategies. However, medical imaging modalities still mostly serve as a secondary or optional component. The next-generation small-scale magnetic robots should at least be compatible with medical imaging modalities to work without direct line-of-sight. In an ideal case, they should also utilize the existing imaging equipments, such as an MRI machine and a US probe, to extend their functionalities catalogue. Recently, healthcare professionals are starting to get involved in the development and deployment of these robots. It is hoped that this collaborative initiative between scientists and doctors will grow stronger with each party bringing unique expertises and perspectives to the table.

In view of the active research activities in the field of small-scale magnetic robotics, this review aims to provide a sense of the context, the landscape, and the state-of-the-art for fellow researchers and engineers. It discusses some exemplar pioneering work that integrated medical imaging modalities into small-scale magnetic robotic systems to various extents. Focus is given to the studies involving MRI, US, X-ray, and fluorescence imaging, due to their popularity and accessibility in existing clinical environments. Although they are not covered in this review, other medical imaging modalities have also been explored to image and track small-scale magnetic robots [103,104]. For example, optical coherence tomography (OTC) was used to track nanometre-scale helical magnetic swimmers in an intact (resected) porcine eye [104]. Photoacoustic imaging was employed to track micron-scale helical magnetic swimmers in a mouse subcutaneous multi-drug-resistant Klebsiella pneumoniae (MDR KP) infection model [103].

## Figures and Tables

**Table 1:** Exemplar studies of small-scale magnetic robots with MRI imaging modality.

Small-Scale Magnetic Robot	Workspace	Reference
Micro/nanorobot with a ferromagnetic core	In vitro and in vivo with tortuous phantom and animal models	2008 [71]
Flagellated magnetotactic bacterium	Carotid artery of a living swine	2009 [72,73]
Ferromagnetic catheter	Renal arteries of rabbits	2015 [77]
Biohybrid helical microswimmers	Rodent stomachs	2017 [75]
Millirobot	Silicone oil pool in a square clear container	2019 [78]
Millimetre-scale Lorentz force actuator module	A square clear plastic container	2020 [74]
Centermetre-scale capsule reversible orientation-locking robot (REVOLBOT)	A synthetic maze embedded in a phosphate-buffered saline (PBS) solution	2021 [79,80]
Neutrophil-based microrobot (“neutrobot”)	In vivo with a glioma-bearing mice	2021 [76]

**Table 2:** Exemplar studies of small-scale magnetic robots with US imaging modality.

Small-Scale Magnetic Robot	Workspace	Reference
Soft sheet robot with a continuously varying sinusoidal magnetization profile	Chamber phantom made with chicken meat	2018 [84]
Jellyfish robot	Water-filled bladder phantom	2019 [85]
Swarm nanorobot	Bovine eyeballs	2019 [86]
Anchoring robot	Tubular phantom	2020 [87]
Tumbling robot	Ex vivo, in vitro, in situ, and in vivo	2020 [88]
Magnetic stem cell spheroid microrobots (MSCSMs)	Bile duct (in vitro and in vivo)	2021 [89]
Nanoparticle microswarm	Near the boundary of porcine coronary artery ex vivo	2021 [90]

**Table 3:** Exemplar studies of small-scale magnetic robots with X-ray imaging modality.

Small-Scale Magnetic Robot	Workspace	Reference
Bullet-shaped millirobot	Vessel phantom	2018 [93]
M-skin millirobot	Stomach of rabbit	2020 [61]
Self-rolled microrobot	Vessel phantom	2021 [94]

**Table 4:** Exemplar studies of small-scale magnetic robots with fluorescence imaging modality.

Small-Scale Magnetic Robot	Workspace	Reference
Bacteria-like microrobotic flagella	Intra peritoneal cavity of mouse	2015 [96]
Biohybrid helical microswimmers	Subcutaneous tissue of nude mice	2017 [75]
Burr-like porous spherical microrobot	Dorsum of nude mice	2018 [97]
Metal-organic-framework-based biomedical microrobots (MOFBOTS)	Microfluidic device	2019 [98]
Fluorescent magnetic spore-based microrobots (FMSMs)	Liquid media	2019 [99]
Cylindrical, hexahedral, helical, and spherical scaffold-type microrobots	In vitro, ex vivo, and in vivo	2019 [100]
Double-helical microswimmer	Petri dish	2020 [101]
